# Workplace Violence Toward Doctors Working in Obstetrics and Gynecology Emergency Units in Khartoum North Locality, Sudan: A Cross-Sectional Study

**DOI:** 10.7759/cureus.46924

**Published:** 2023-10-12

**Authors:** Eithar M Ali, Walaa A Mohammed, Duaa S Mahmoud, Tibyan A F. Kheiralla, Eman A Nasrallah, Khansaa M Elfadul, Tawheed Abdelfatah Hamza Ahmed, Halima I Hussein, Ibrahim H Elkhidir, Mohamed S Muneer

**Affiliations:** 1 Faculty of Medicine, University of Khartoum, Khartoum, SDN; 2 Faculty of Medicine, University of Khartoum, Doha, QAT; 3 Department of Medicine, Ribat University Hospital, Khartoum, SDN

**Keywords:** workplace violence, khartoum north locality, africa, sudan, emergency departments, obstetrics and gynecology, wpv

## Abstract

Background and objectives: Workplace violence (WPV) is any action, incident, or behavior that deviates from appropriate conduct and results in a person getting assaulted, threatened, harmed, or injured at work. This research aimed at studying the current state of WPV among doctors working in obstetrics and gynecology (OBGYN) emergency departments (EDs) in Khartoum north locality (KNL), Sudan Methods: A descriptive cross-sectional study that included 128 doctors from six governmental hospitals in KNL. A self-administered questionnaire assessing the prevalence and outcomes of WPV was distributed. The descriptive statistics and frequency tables were generated using the Statistical Package for Social Sciences (SPSS) version 21 (IBM Corp., Armonk, NY). Results: The respondents’ mean age was 28.3±6.6 years (range: 21-70 years). Approximately half of the respondents (49.2%) experienced WPV. Verbal WPV was the most common type (93.3%), followed by physical (10%) and sexual (3.2%) type. Patients’ relatives and friends are the most common group to commit WPV (92.9%). Night shifts were the time most WPV (58.6%) took place. The effect of WPV on respondents was mainly psychological (95.8%) compared to physical (4.2%). Conclusion: WPV prevalence among health care workers (HCWs) working in the OBGYN EDs is alarming with detrimental effects. Evaluating the current state of WPV, outcome, and associated factors will help not only address the current problem but also guide future related research.

## Introduction

The World Health Organization (WHO) defines violence as “the intentional use of physical force or power, threatened or actual, against oneself, another person, or a group or community that either results in or has a high likelihood of resulting in injury, death, psychological harm, mal-development, or deprivation” [1]. Workplace violence (WPV) is defined by the International Labor Organization (ILO) as “any action, incident, or behavior that departs from reasonable conduct in which a person is assaulted, threatened, harmed, or injured in the course of, or as a direct result of, his or her work” [2]. Worldwide, healthcare workers (HCWs) are at high risk of WPV with 8% to 38% of HCWs having experienced physical WPV at some point in their careers [3]. It has been reported that from 70% to 74% of the cases of WPV were reported in healthcare settings and social service settings in the period between 2011 and 2014 [4]. Africa has ranked the fourth continent according to the number of WPV among HCWs [5]. In South Africa, the WPV incidence is 61% [6]. The situation is described as endemic in sub-Saharan Africa [7]. In Sudan, a study among HCWs showed that 50% of participants experienced WPV [8]. Within the different hospital departments, emergency departments (EDs) reported the highest number of WPV incidents [9-12].

WPV in healthcare settings has detrimental effects on HCWs such as increased absence from work, decreased work performance and satisfaction, and poor mental health, all resulting in suboptimal patient care [13]. Moreover, suboptimal patient care and not high-performing HCWs create a vicious circle, which perpetuates WPV [14].

The increased WPV toward HCWs in Sudan is alarming. The Republic of Sudan implemented a doctors’ protection law that included a penalty of up to 10 years in prison. We hypothesized that obstetrics and gynecology EDs (OBGYN-EDs) in the Sudanese healthcare system had a higher incidence of WPV due to inadequate facilities and overcrowding. To our knowledge, this is the first study evaluating WPV among HCWs in OBGYN-EDs in Khartoum north locality (KNL), Sudan. It aims to study types of WPV and variations regarding age, gender, marital status, and job description. Also, it aims to determine the timing of WPV occurrence, perpetrators, and the impact of WPV on doctors.

## Materials and methods

Study design and participants

A descriptive cross-sectional, OBGYN-EDs-based study was conducted in KNL, one of the seven localities of Khartoum State, Sudan. The included hospitals were selected excluding hospitals with no OBGYN departments. The total number of doctors working in the included OBGYN-EDs was 252, ranging from juniors (house officers) and seniors (resident physicians, specialists, and consultants).

Sample size and data collection

The study population was doctors who were working in OBGYN-EDs and there were a total of 252 doctors. The study sample size was calculated using a sample size calculator [15]. The calculated sample size of 153 was deemed appropriate for our confidence level of 95%. A total of six hospitals were randomly selected. Out of 153 doctors, 128 responded, hence achieving an 83.7% response rate.

Ethical consideration: approval and informed consent

The study was approved by the ethical committee of the community medicine department at the faculty of medicine, university of Khartoum. Informed consent was obtained from each respondent.

Statistical analysis

Data was collected using a pre-coded and self-administered questionnaire. The questionnaire was formulated based on a thorough review of the relevant literature and comprised of socio-demographic, work-related, and violence-related questions (for any violent incident in the past two months). Also, it included questions on the effect of violence and the response of doctors. Collected data were entered into a Microsoft Excel sheet (Albuquerque, New Mexico, USA), then cleaned and audited. Then for analysis, data was transferred into the Statistical Package for Social Sciences (SPSS, version 21; IBM Corp., Armonk, NY). Descriptive statistics and frequency tables were generated. Inferential statistics were applied to test for any statistically significant difference based on variable types under comparison. A statistical significance level at a P-value of 0.05 was chosen [16].

## Results

The study response rate was 82.6% (128/155). The mean age of the respondents was 28.3±6.6 years (range 21-70 years). As shown in Table 1, most of the respondents were females (71.1%) while 37 (28.9%) were males. Approximately a third of all respondents were married, 44 (34.4%), with the remaining 83 (64.8%) reporting single social status. Regarding the level of experience/training and job description, 58 (45.3%) were juniors (house officers) and 70 (54.7%) were seniors (resident physicians, specialists, and consultants). The aforementioned socio-demographic factors and the job description showed no statistically significant difference with regard to the incidence of WPV (Table 2). Almost half of the respondents, 63 (49.2%), experienced WPV. Of those respondents, 56 (93.3%) experienced verbal violence, followed by six (10%) physical violence and two (3.2) sexual violence. Most of these incidents, 52 (92.9%), were committed by co-patients (patients' relatives and friends), with the remainder, four (7.1%) incidents committed by the patients themselves. Regarding the distribution of WPV occurrence between day and night shifts, most of the incidents took place during night shifts, accounting for 34 (58.6%) of the total reported incidents (Table 3).

**Table 1:** Socio-demographic data and job description of respondents Values are presented as n (%)

		Frequency	Percentage (%)
Gender	Male	37	28.9
(n=128)	Female	91	71.1
Age groups	21-30	87	78.4
(n=111)	31-40	18	16.2
	41-50	2	1.8
	51-60	3	2.7
	61-70	1	0.9
Marital status	Married	44	34.4
(n=128)	Single	83	64.8
	Divorced	1	0.8
	widowed	0	0
Job description	Junior	58	45.3
(n=128)	Senior	70	54.7

**Table 2:** Prevalence of WPV among respondents and association of socio-demographic data with WPV *P-value has been calculated using the Chi-square test WPV - Workplace Violence

Question		Option	Experienced violence before		P-value*
			Yes	No	
Age	21-30		44	43	0.469
(n=128)	31-40		8	10	
	41-50		2	0	
	51-60		1	2	
	61-70		0	1	
Gender	Male		17	20	0.637
(n=110)	Female		46	45	
Job description (n=128)	Junior		28	30	0.987
	Senior		35	35	
Marital status (n=128)	Married		22	22	0.612
	Single		41	42	
	Divorced		1	0	
	widowed		0	0	

**Table 3:** Types, time, consequences, and impact of WPV WPV - Workplace Violence

Questions	Options	Answers	
Have you experienced verbal, physical, or sexual WPV in the past two months? (n=128)	Yes	63 (49.2%)	
	No	65 (50.8%)	
	What type of WPV? (multiple answers allowed) (n=63)		Yes	
Verbal		56 (93.3%)	
Physical		6 (10%)	
Sexual		2 (3.2%)	
WPV was committed by? (n=56)	Patient	4 (7.1%)	
	Co-patient (patient's relatives and friends)	52 (92.9%)	
When did you experience violence? (n=58)	Day	24 (41.4%)	
	Night	34 (58.6%)	
How many times did you experience violence? n=(45)	Once	22 (40.7%)	
	More than one	23 (51.1%)	
Are you affected by this violence? (n=63)	Yes	51 (81.0%)	
	No	12 (19.0%)	
If yes, which type of effect? (n=48)	Psychological	46 (95.8%)	
	Physical	2 (4.2%)	
Were you absent from work after experiencing WPV? (n=61)	Yes	17 (27.9%)	
	No	44 (72.1%)	
What was your immediate action?(n=63)	Responded verbally	26 (41.3%)	
	Did not respond	37 (58.7%)	
Did you report this violence? (n=63)	Yes	28 (44.4%)	
	No	35 (55.6%)	
To whom you reported this violence? (n=28)	Police	7 (25.0%)	
	Your supervisor	17 (60.7%)	
	Family member	4 (14.3%)	
What punishment was given to the attacker? (n=28)	Verbal	1 (3.6%)	
	Prison	1 (3.6%)	
	Nothing	26(92.9)	

Effect of WPV and the response of doctors toward violence

WPV affected 51 (81%) of victims with 46 reporting psychological effects and two reporting physical effects. Over a quarter of the victims, 17 (27.9%) reported the need to be absent from work due to WPV. Twenty-six (41.3%) victims verbally reacted to the violence at the time of the incident, and the remainder 37 (58.7%) did not. Only 28 (44.4%) of the victims reported the WPV occurrence, of which 17 (60.7%) reported it to their supervisors, seven (25%) to the police, and the remaining to their family members. Among those reporting the WPV incidents, 26 (92.9%) reported the perpetrators suffered no consequences. Almost half of the respondents reported experiencing multiple WPV occurrences as shown in Table 3.

## Discussion

WPV among HCWs is a problem globally [6]. Our study found no association between the socio-demographic characteristics of the respondents and WPV. Also, in our study, more than half of the respondents experienced violence in the past two months, in the form of verbal, physical, and/or sexual violence. Doctors providing services at night are at an increased risk of encountering WPV. Additionally, co-patients (patients' relatives and friends) had a higher likelihood of initiating WPV than patients. Half of the respondents reported being affected physically or psychologically, and less of them were absent from work. In dealing with WPV, most victims did not react. Moreover, less than half of respondents reported the violence to their boss, police, or family members.

Almost half of our respondents (49.2%) experienced violence in the previous two months. This is similar to previous studies conducted in Myanmar (47.6%) [17], and India (47.02%) [18]. It is worth mentioning that some other studies had reported a slightly higher WPV incidence than this study, as 65% in Sudan [8], 64% in Ethiopia [19], and 59.7% in Egypt [20]. WPV may be influenced by a range of factors, including inadequate communication, lack of resources, and lack of cultural diversity. In addition, long waiting times and the mismatch between the expectations of patients and the actual care predispose to violence.

Most of our respondents were female, accounting for 71.1% of all respondents. This study found socio-demographic characteristics (age, gender, job description, and marital status) had no statistically significant association with WPV incidence. Similar findings were reported by Boafo et al. in Ghana [21]. However, another study found males had a greater risk than females of experiencing WPV in Sudan [8]. More WPV incidents occurred during night shifts, similar to a prior study done among doctors in Sudan [8] and other studies done among HCWs in Bahrain [22] and Egypt [20]. This may be attributed to reduced security staffing or having more critical cases with anxious patients and co-patients during night shifts.

Verbal violence was found to be the most common type of violence in the present study (93.3%). This is consistent with studies in Egypt (86.1%) [23] and India (87.3%) [18]. Verbal violence usually precedes physical one and this highlights the importance of training HCWs in de-escalation techniques to prevent progression of the verbal violence into physical one [24]. The perpetrators in Karachi, Pakistan, were mainly co-patients [8], which aligns with our findings. It is also consistent with other studies [13,20,21]. WPV has had various impacts on the HCWs whether physical, psychological, or work-performance-related aspects. The impact may last for a brief or prolonged period. The psychological effect may manifest as increased anxiety, fear, depression, and/or altered mental health [13]. Notably, the present study found most doctors were mentally affected, similar to a study conducted in Italy [25]. This leads to a decrease in productivity, an increase in turnover rate, and higher rates of absenteeism. Moreover, it results in elevated counseling expenses, a decline in staff humor, and a reduction in quality of life [26-29]. Additionally, our findings revealed that many respondents did not report the incident. This behavior may be explained by various factors, previous experiences of injustice to doctors [30] and fear of losing one’s job [14]. Of note, stigmatization related to sexual violence in the local cultural context may contribute to this behavior. Unfortunately, in most of the reported violence, the perpetrators suffered no consequences.

Study limitations

The limitation of this study is that it studies WPV from a doctor’s perspective only, and WPV can happen to other HCWs in the hospitals such as nurses, excluding them may underestimate the magnitude of the problem. The prevalence would differ if participants were asked to recall WPV in the past 12 months instead of the most recent two.

## Conclusions

WPV is an important issue facing HCWs in hospitals, with its great effects on their lives and the services provided to patients. Through this research, we would like to draw attention to the existence of Sudan’s law on the protection of doctors, medical staff, and health established on May 29, 2020. Unfortunately, it has not been activated or applied by the authorities till now. WPV problem-solving in our perspective could be made by applying Sudan’s law by the stakeholders in all hospitals throughout the country and encouraging HCWs to report the event. Training for recognizing and adequately managing and de-escalating violence, especially verbal ones, by health professionals, and ensuring the presence of well-trained and adequate security personnel, especially during the night shifts may improve the situation. In addition, doctors should receive training in communication skills, including breaking bad news. Equally important raising awareness on this issue among the public and HCWs will help decrease WPV incidents, if not preventing them. Future research studies addressing WPV occurrence from other HCWs’ perspectives in Sudan other than doctors are warranted. Also, studies evaluating the effectiveness of the different interventions to mitigate and prevent WPV are needed.
